# Sustained acceleration of soil carbon decomposition observed in a 6-year warming experiment in a warm-temperate forest in southern Japan

**DOI:** 10.1038/srep35563

**Published:** 2016-10-17

**Authors:** Munemasa Teramoto, Naishen Liang, Masahiro Takagi, Jiye Zeng, John Grace

**Affiliations:** 1 Center for Global Environmental Research, National Institute for Environmental Studies, Tsukuba, Ibaraki 305-8506, Japan; 2 Faculty of Agriculture, University of Miyazaki, 11300 Tano-cho, Miyazaki 889-1702, Japan; 3 Institute of Ecology and Resource Management, University of Edinburgh, Edinburgh EH9 3JU, UK

## Abstract

To examine global warming’s effect on soil organic carbon (SOC) decomposition in Asian monsoon forests, we conducted a soil warming experiment with a multichannel automated chamber system in a 55-year-old warm-temperate evergreen broadleaved forest in southern Japan. We established three treatments: control chambers for total soil respiration, trenched chambers for heterotrophic respiration (*R*_h_), and warmed trenched chambers to examine warming effect on *R*_h_. The soil was warmed with an infrared heater above each chamber to increase soil temperature at 5 cm depth by about 2.5 °C. The warming treatment lasted from January 2009 to the end of 2014. The annual warming effect on *R*_h_ (an increase per °C) ranged from 7.1 to17.8% °C^−1^. Although the warming effect varied among the years, it averaged 9.4% °C^−1^ over 6 years, which was close to the value of 10.1 to 10.9% °C^−1^ that we calculated using the annual temperature–efflux response model of Lloyd and Taylor. The interannual warming effect was positively related to the total precipitation in the summer period, indicating that summer precipitation and the resulting soil moisture level also strongly influenced the soil warming effect in this forest.

Soil respiration (*R*_s_) consists of root respiration (autotrophic respiration) and heterotrophic respiration (*R*_h_) by soil-dwelling microbiota, and it is the second largest carbon flux in terrestrial ecosystems. The global *R*_s_ was estimated at 98 ± 12 GtC in 2008^1^, and *R*_h_ was estimated to be 57.1 GtC^2^, more than half of *R*_s_.

Enzymatic activity by the soil microbiota is expected to increase with temperature, and previous studies have expressed the temperature sensitivity of *R*_h_ by using a simple exponential function^3^, *Q*_10_, or other, more exact functions^4^,^5^. Many modelling studies have attempted to represent changes in the carbon cycle under global warming using a constant value of *Q*_10_^6^,^7^,^8^,^9^. However, a recent study by Todd-Brown *et al*.^10^ used several CMIP5 Earth System Models and showed that *Q*_10_ values for global soil carbon decomposition rates ranged from 1.45 to 2.61. The exponential function for *Q*_10_ implies that global *R*_h_ will rise dramatically with even a small increase in global temperatures. According to the worst-case RCP 8.5 scenario in the 5th IPCC report^11^, the world’s temperature may increase by 2.6 to 4.8 °C by the end of the 21st century. Even though the global terrestrial ecosystem is now estimated to represent a carbon sink of 2.4 ± 0.7 GtC^12^, rising temperatures are likely to convert much of this carbon sink into a carbon source due to the increased *R*_h_ that will occur under global warming^13^.

On the other hand, some studies have shown no evidence of a long-term exponential increase of soil CO_2_ emission in response to global warming^14^,^15^,^16^,^17^. The decreased or weaker-than-expected soil warming effects can be explained by several factors, including differences in the quantity of soil organic carbon (SOC)^18^,^19^, precipitation and soil moisture^20^, thermal adaptation of microbiota due to shifts in enzymatic activity^21^, and changes in the microbial species composition^22^,^23^,^24^ and biomass^25^.

Using models with several soil carbon pools, previous studies have explained the decreased stimulatory effect of soil warming by rapid depletion of labile carbon fractions^18^,^19^. However, the temperature sensitivity of non-labile organic carbon fractions is also a serious concern for determining the long-term response of *R*_h_ to warming^19^,^26^. Therefore, the quantity of SOC appears to be critical for determining the long-term response of *R*_h_ to warming.

Stimulation of *R*_h_ by soil warming is also closely related to precipitation and soil moisture because soil warming tends to decrease the soil moisture content^27^. Water deficits decrease *R*_h_ and *Q*_10_ by suppressing microbial activity through the inhibition of solute diffusion and a decrease of substrate availability^28^,^29^. Thus, under water-deficit conditions, soil moisture could become the primary factor that will govern soil carbon decomposition, and the soil warming effect might not be apparent or might even suppress CO_2_ emission due to drying of the soil^20^,^30^.

To understand the long-term response of *R*_h_ to warming and the factors that affect the response, it is necessary to use long-term experiments. Recently, observations over long periods have begun to appear^16^,^17^,^31^,^32^,^33^,^34^, but the observed long-term warming effect has been inconsistent. More observations and interpretations are therefore urgently needed.

In the present work, we focused on a warm-temperate forest in southern Japan, where the monsoon climate is humid, drought is infrequent, and the soil is relatively rich in carbon^35^,^36^,^37^. To examine the trend of stimulatory soil warming effect on *R*_h_ under these humid and SOC-abundant conditions, and to analyse how the effects of interactions among various factors affected the response, we conducted a soil warming experiment. Most previous soil warming experiments were conducted in grasslands^15^,^38^,^39^, boreal coniferous forests^16^,^18^,^40^, and cool-temperate forests^17^,^33^,^41^. Our study therefore complements this previous research by providing new data about the long-term soil warming effect in a warm-temperate forest.

We used a multichannel, automated chamber measurement system to collect soil CO_2_ efflux data continuously under an artificially warmed environment in a warm-temperate evergreen broad-leaved forest site on Kyushu, in southern Japan. Continuous observation with a high-resolution dataset made it possible to detect the influences of short-term environmental changes on the soil CO_2_ efflux, thereby providing data suitable for accurate estimation of a potential long-term warming effect. This is important, because soil warming experiments with such continuous observations have been rare in the world warmer region. To our knowledge, this is the first experiment that examined the effect of soil warming on *R*_h_ using long-term continuous measurements in a warm-temperate forest.

## Results

### Soil carbon and nitrogen

The SOC and total nitrogen (TN) contents in the upper 30 cm of the soil near chambers were averaged 9.92 ± 0.48 kg m^−2^ and 0.69 ± 0.03 kg m^−2^, respectively. The C:N ratio of the upper 30 cm of the soil was 14.2. The SOC in the upper 5 cm was estimated to be 2.23 kg m^−2^ in trenched treatment (*R*_h_), and 2.09 kg m^−2^ in warmed trenched treatment (*R*_hw_) after 6 years of warming treatment in the end of December 2014. No significant warming effect on SOC content was confirmed.

### Seasonal variability of the CO_2_ flux and the influence of soil temperature and moisture

**Figure 1:**
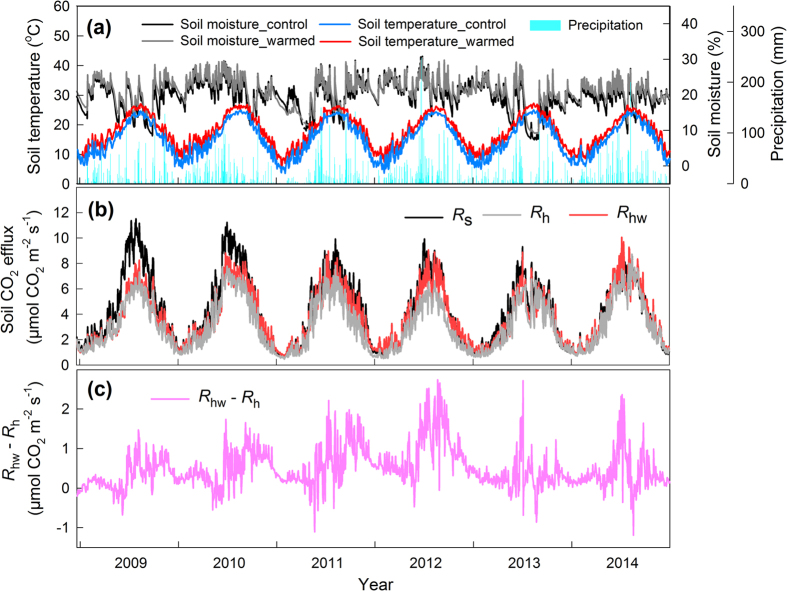
The seasonal changes of (**a**) soil temperature (at a depth of 5 cm) and soil moisture content (at a depth of 10 cm), (**b**) soil CO_2_ efflux (*R*_s_, soil respiration; *R*_hw_ and *R*_h_, heterotrophic respiration in the warmed and control soil, respectively), and (**c**) the difference in efflux between the warmed and control treatments (i.e., *R*_hw_−*R*_h_).

We obtained 6 years of continuous soil CO_2_ efflux data (*F*_c_). *F*_c_ increased with increasing soil temperature in all treatments (Fig. 1a,b). The mean *F*_c_ values during the whole study period were 4.13 ± 0.36 μmol CO_2_ m^−2^ s^−1^ for *R*_s_, 3.14 ± 0.08 μmol CO_2_ m^−2^ s^−1^ for *R*_h_, and 3.63 ± 0.16 μmol CO_2_ m^−2^ s^−1^ for *R*_hw_. The difference in efflux between *R*_hw_ and *R*_h_ usually peaked during the summer (July to September), but it decreased or even became negative when the soil moisture level was low during the summer or at the end of the spring (from late May to early June, Fig. 1c). During the whole measurement period, *R*_h_ accounted for 76.0% of *R*_s_.

**Figure 2:**
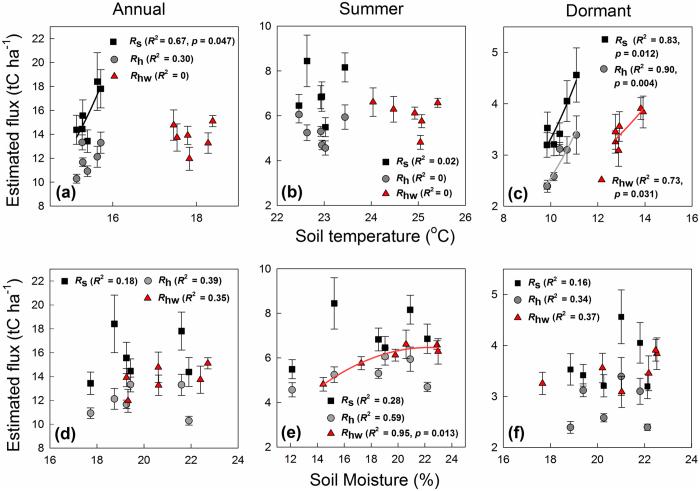
Relationships between the estimated *F*_c_ values (*R*_s_, soil respiration; *R*_hw_ and *R*_h_, heterotrophic respiration in the warmed and control soil, respectively) and the (**a**,**b**,**c**) average soil temperature at a depth of 5 cm and (**d**,**e**,**f**) soil moisture content at a depth of 10 cm for the (**a**,**d**) annual period, (**b**,**e**) summer, and (**c**,**f**) dormant period. ‘Summer’ means from July to September and ‘dormant period’ means from January to April and November to December of each year. Values are means ± SEM.

The estimated total annual carbon flux was 15.7 ± 1.4 tC ha^−1^ for *R*_s_ (with a range from 13.4 to 18.4 tC ha^−1^ during the study period), 11.9 ± 0.3 tC ha^−1^ for *R*_h_ (ranging from 10.3 to 13.3 tC ha^−1^), and 13.8 ± 0.6 tC ha^−1^ for *R*_hw_ (ranging from 12.0 to 15.1 tC ha^−1^). The estimated carbon flux *R*_hw_ increased significantly due to the warming treatment both annually (*p* = 0.033) and seasonally (during the dormant period, *p* = 0.028) compared with *R*_h_, but the difference was not significant during the growing period (from May to October) or the summer. Figure 2 shows the seasonal relationships between the estimated *F*_c_ and the soil temperature (Fig. 2a–c) and the soil moisture (Fig. 2d–f). This analysis revealed significant relationships between soil temperature and *F*_c_ in the dormant period (*p* < 0.050, *R*^2^ > 0.7, Fig. 2c). There was no significant relationship between soil temperature and each carbon flux in the summer, when the temperature range was relatively small (Fig. 2b). On the other hand, the relationship between soil moisture and *F*_c_ was only significant for *R*_hw_ during the summer (*p* = 0.013, *R*^2^ = 0.95, Fig. 2e). There was no relationship between soil moisture and *F*_c_ in the dormant period in all treatments (Fig. 2f).

### Temperature response of *F*_c_

**Figure 3:**
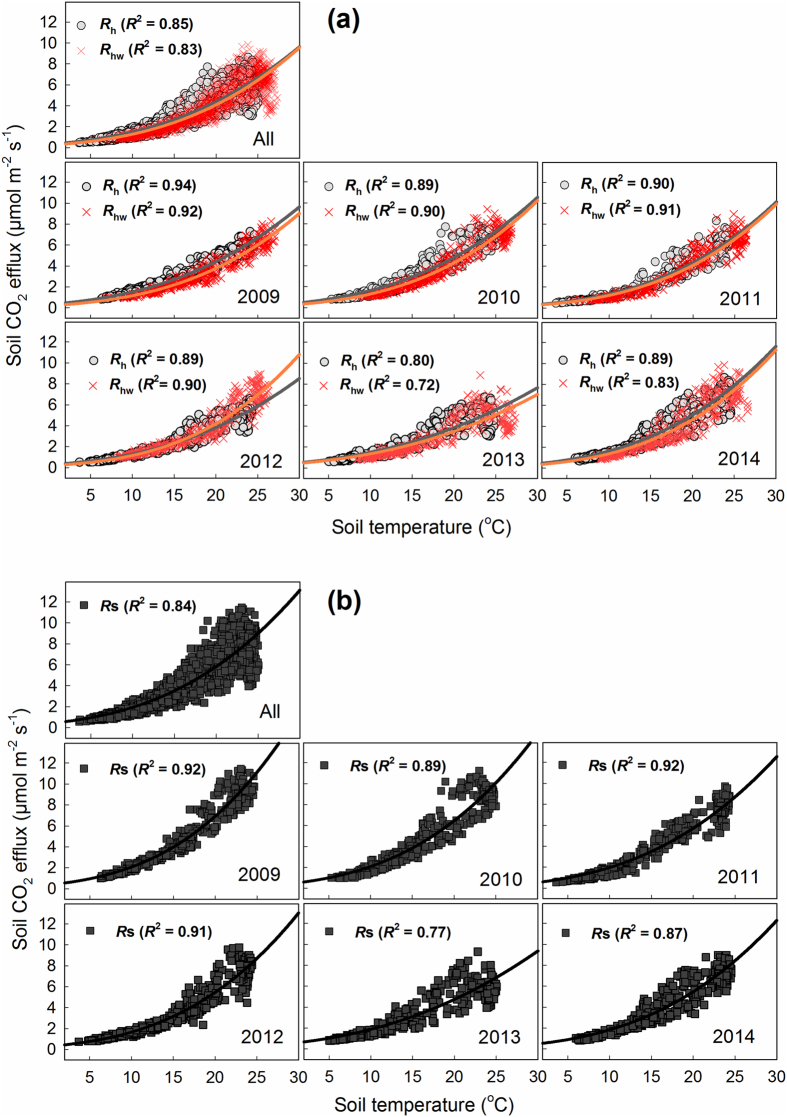
Temperature response of soil CO_2_ efflux for (**a**) heterotrophic respiration in the control (*R*_h_) and warmed (*R*_hw_) treatments and (**b**) soil respiration (*R*_s_) in each year. Soil temperature was measured at a depth of 5 cm. Regression lines are only presented for statistically significant relationships (*p* < 0.0001). The equation of Lloyd and Taylor (Equation 4) was used for the fitted curve.

*F*_c_ increased exponentially with increasing soil temperature in both the control and the warming treatment in all years (*p* < 0.0001, *R*^2^ > 0.80, Fig. 3a,b), with the exception of 2013 (for which the relationship was significant, but weaker, with *R*^2^ > 0.70).

### Response of *F*_c_ to soil moisture

**Figure 4:**
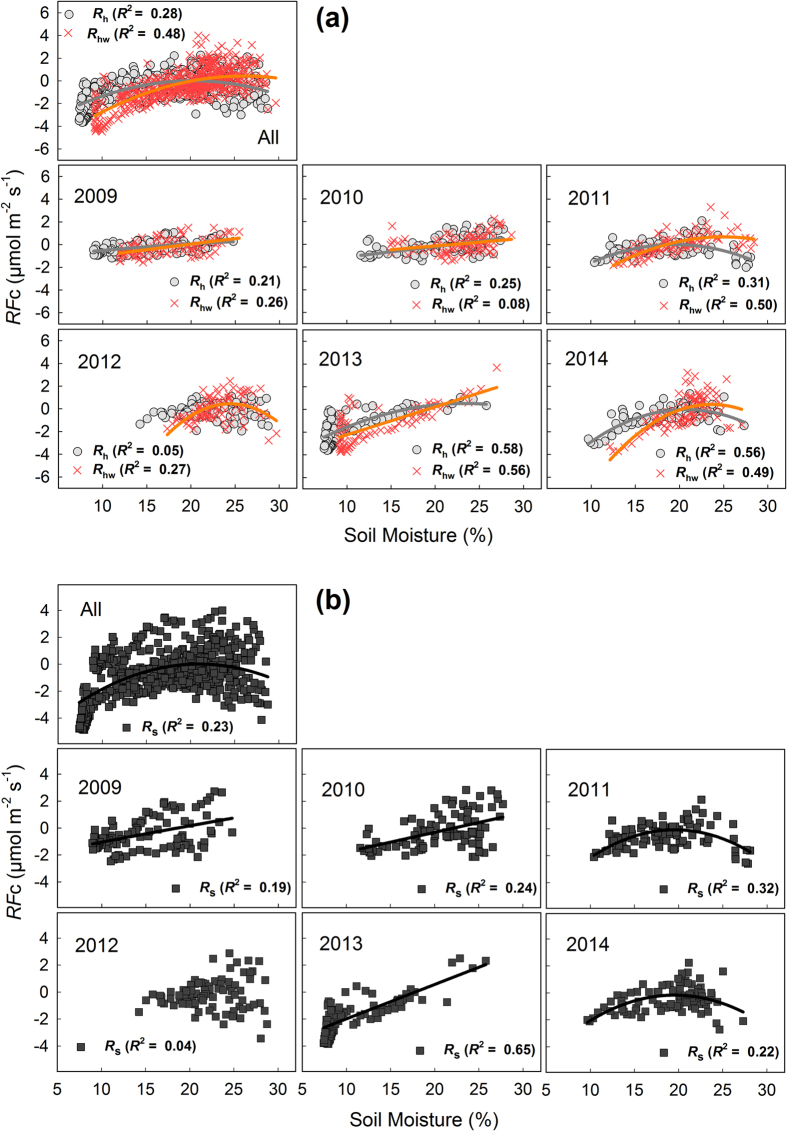
Moisture response of the temperature-normalized *F*_c_ (*RF*_c_) from the fitted curves in Figure for (**a**) heterotrophic respiration in the control (*R*_h_) and warmed treatment (*R*_hw_) and (**b**) soil respiration (*R*_s_) from July to September in each year. Regression lines are only presented for statistically significant relationships (*p* < 0.01). Soil moisture content was measured at a depth of 10 cm.

We analysed the relationship between *F*_c_ and soil moisture from July to September using temperature-normalized *F*_c_ (*RF*_c_: the residuals of measured *F*_c_ and predicted values of *F*_c_ using Equation 4) (Fig. 4a,b). Usually, the relationship was a concave-down curve (i.e., exhibited a maximum value). However, the relationship was relatively weak when we used *RF*_c_ data from all 6 years (*p* < 0.0001, *R*^2^ = 0.23 for *R*_s_, 0.28 for *R*_h_, 0.48 for *R*_hw_, Fig. 4a,b). There was no significant relationship between *RF*_c_ and soil moisture in the summer of 2012 for *R*_s_ and *R*_h_. However, we detected a moderately strong relationship in 2013 (*p* < 0.0001, *R*^2^ = 0.65 for *R*_s_, 0.58 for *R*_h_, 0.56 for *R*_hw_, Fig. 4a,b) when summer precipitation was the lowest in 6 years.

### Temperature sensitivity of *Q*_10_

**Figure 5:**
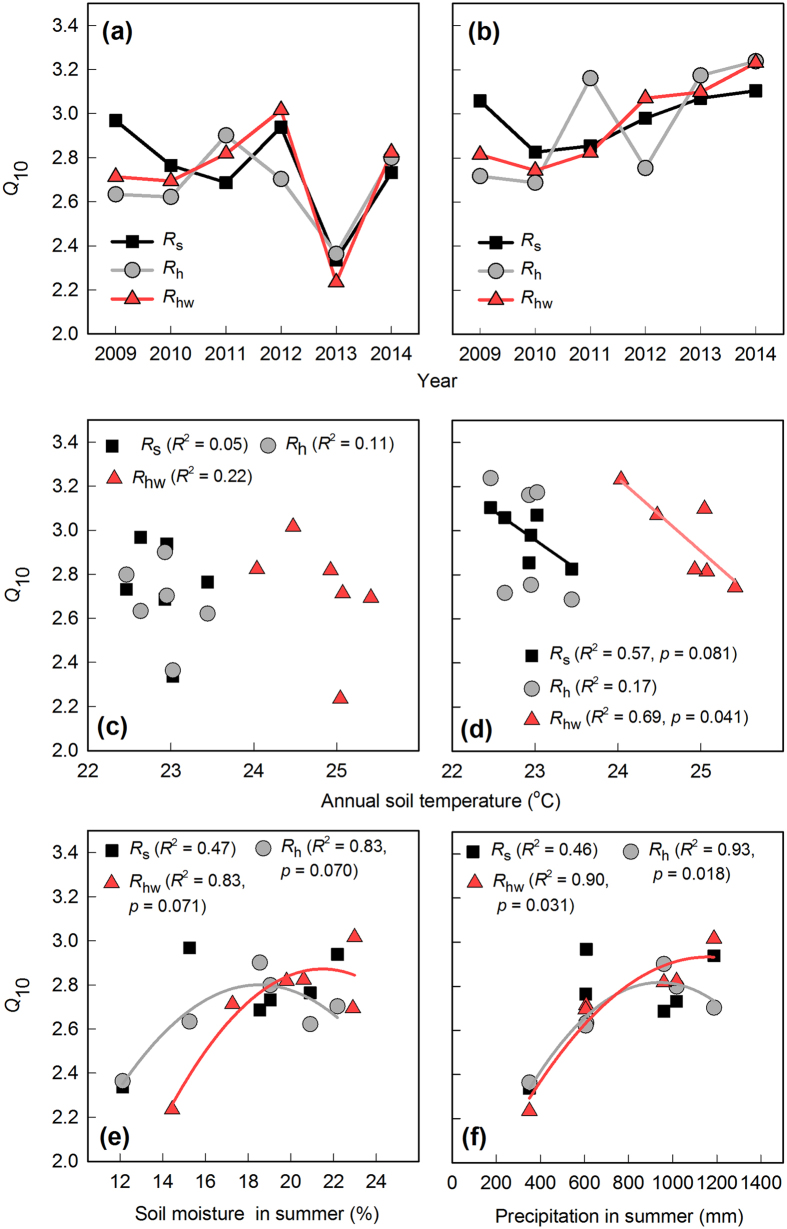
Annual trends of (**a**) *Q*_10_ (raw-*Q*_10_) and (**b**) soil-moisture-normalized *Q*_10_ (normalized-*Q*_10_). Relationships between the mean annual temperature and (**c**) raw-*Q*_10_ and (**d**) normalized-*Q*_10_. Relationships between (**e**) soil moisture in the summer (from July to September) and raw-*Q*_10_ and between (**f**) precipitation in summer and raw-*Q*_10_. The normalized-*Q*_10_ was calculated from *F*_c_ data only when the soil moisture content was within the range of annual average ± 1 SD. Regression lines are only presented for statistically significant relationships (*p *< 0.1).

We analysed the temperature sensitivity of the carbon efflux (*Q*_10_). To remove the effect of soil moisture from this *Q*_10_ analysis, we filtered the *F*_c_ data to obtain values when soil moisture was within the range for the annual average of soil moisture ± 1 SD (i.e., the soil-moisture-normalized value). Figure 5a,b show the annual trends of *Q*_10_ values for the all measured data (raw-*Q*_10_) and soil-moisture-normalized data (normalized-*Q*_10_), respectively. The mean raw-*Q*_10_ value for *F*_c_ was 2.73 (ranged from 2.34 to 2.97) for *R*_s_, 2.65 (ranged from 2.36 to 2.90) for *R*_h_, and 2.66 (ranged from 2.23 to 3.02) for *R*_hw_. There was no significant difference in this overall average raw-*Q*_10_ between *R*_h_ and *R*_hw_. The decrease of raw-*Q*_10_ in 2013 was remarkable in all treatments (Fig. 5a). On the other hand, we found that the normalized-*Q*_10_ value increased to 3.00 (ranged from 2.83 to 3.10) for *R*_s_, 2.88 (ranged from 2.69 to 3.24) for *R*_h_, and 2.92 (ranged from 2.74 to 3.23) for *R*_hw_ (Fig. 5b). The annual normalized-*Q*_10_ became more stable, and the decrease in the raw-*Q*_10_ in 2013 disappeared. Similarly, there was no significant difference in the mean normalized-*Q*_10_ between *R*_h_ and *R*_hw_.

We found no significant relationship between the annual raw-*Q*_10_ and soil temperature (Fig. 5c). However, we found marginally significant and significant negative relationships between soil temperature and the normalized-*Q*_10_ for *R*_s_ (*p* = 0.081, *R*^2^ = 0.57) and *R*_hw_ (*p* = 0.041, *R*^2^ = 0.69), respectively (Fig. 5d). On the other hand, we found marginally significant concave-down relationships between summer soil moisture and the annual raw-*Q*_10_ (Fig. 5e) for *R*_h_ (*p* = 0.070, *R*^2^ = 0.83) and for *R*_hw_ (*p* = 0.071, *R*^2^ = 0.83). In addition, we found significant and slightly stronger concave-down relationships between the total summer precipitation and the annual raw-*Q*_10_ (Fig. 5f) for *R*_h_ (*p* = 0.018, *R*^2^ = 0.93) and for *R*_hw_ (*p* = 0.031, *R*^2^ = 0.90).

### Effect of warming on *R*_h_

**Figure 6:**
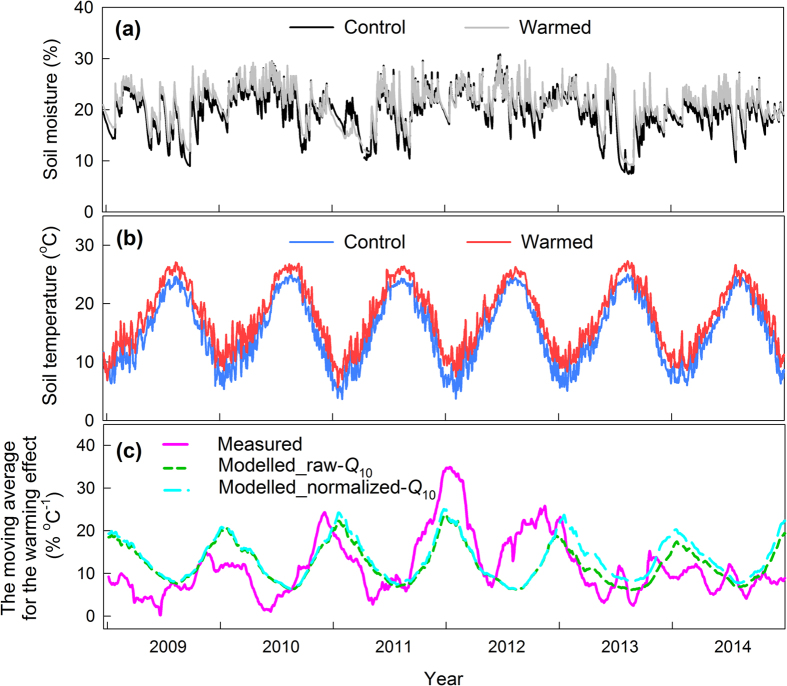
Seasonal variation of (**a**) soil moisture content, (**b**) soil temperature, and (**c**) the 31-day moving average for the warming effect. Raw-*Q*_10_ model values were estimated from the annual temperature-response equation of Lloyd and Taylor (Equation 4) using the all measured data without accounting for soil moisture anomalies. Normalized-*Q*_10_ model values were estimated from the equation that only included *F*_c_ data from times when the soil moisture was within the range of annual average ± 1 SD.

Our measurements showed a clear seasonal pattern in the warming effect (Fig. 6). Overall, the warming effect increased from September to annual maximum peak in winter (from the middle of November to January), and decreased after the peak. The warming effect was kept low level from May to August. During the 6 years of measurement, the maximum peak of the warming effect was in early January 2012, and the effect decreased thereafter. We calculated modelled warming effect using raw-*Q*_10_ model and normalized-*Q*_10_ model. The modelled warming effect showed the same basic seasonal pattern: large in winter and small in summer (Fig. 6).

**Figure 7:**
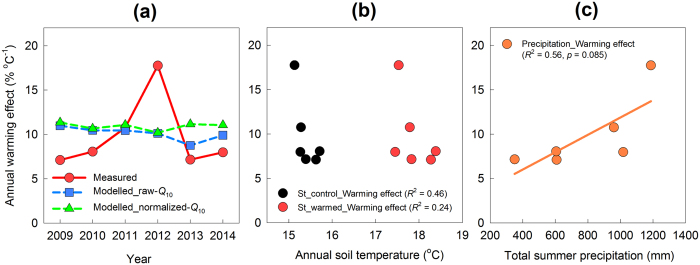
(**a**) Annual variation in the warming effect on soil CO_2_ efflux (measured values, and values estimated using the raw-*Q*_10_ model and the normalized-*Q*_10_ model). (**b**) Relationships between the mean annual soil temperature (control and warmed) and the annual warming effect. (**c**) Relationships between the total summer precipitation (from July to September) and the annual warming effect. Regression lines are only shown for statistically significant relationships (*p* < 0.1).

The annual warming effect was 7.1% °C^−1^ in 2009, 8.1% °C^−1^ in 2010, 10.8% °C^−1^ in 2011, 17.8% °C^−1^ in 2012, 7.2% °C^−1^ in 2013, and 8.0% °C^−1^ in 2014 (Fig. 7a). The annual variation in the warming effect was obvious, and the measured warming effect peaked in 2012, as shown in Fig. 6. However, the overall average warming effect for the study period was 9.4% °C^−1^, which is very close to the modelled warming effects (10.1% °C^−1^ for the raw-*Q*_10_ model, 10.9% °C^−1^ for the normalized-*Q*_10_ model). The interannual variation of the modelled warming effect was much smaller than that of the measured effect, ranging from 8.8 to 11.0% °C^−1^ in the raw-*Q*_10_ model and from 10.2 to 11.4% °C^−1^ in the normalized-*Q*_10_ version. The difference between the two versions of the modelled warming effect was biggest in 2013 (2.4% °C^−1^), but was minor in the other years (0.1–1.2% °C^−1^) as was shown in Figs 6 and 7a. There was no significant relationship between the annual warming effect and the annual mean soil temperature (Fig. 7b). On the other hand, we found a marginally significant positive relationship between the annual warming effect and the total summer precipitation (*p* = 0.085, *R*^2^ = 0.56, Fig. 7c).

## Discussion

The stimulatory effect of soil warming was maintained in all years, with no indication of any decrease or disappearance of the warming effect. Even though we observed strong interannual variation of the soil warming effect, its overall average value was 9.4% °C^−1^, which is very close to the values estimated by the traditional temperature response model. Regarding the magnitude of the soil warming effect on *R*_h_, Wang *et al*.^42^ estimated values of 13.5, 3.5, and 10.5% °C^−1^ for forest, grassland, and an overall mean of 50 ecosystems (after 1 to 19 years of warming), and suggested that no apparent thermal adaptation had occurred after 4 years of warming. Our result agrees well with their report.

The sustainability of the warming effect in the present study appears to depend on the abundance of SOC. The amount of SOC (to a depth of 30 cm) at our study site (9.92 kg m^−2^) was slightly larger than the estimated average SOC for Japanese forest soils (9.0 kg m^−2^)^35^, which in turn was 1.7 times the world average. From this perspective, the SOC content was high at our forest site. Some authors have explained a decreasing trend for the warming effect after several years of warming by hypothesizing depletion of the labile C pools^18^,^19^. Indeed, in a warming experiment that used the same protocol with our study conducted in a mixed-forest in an old-peatland in northern Japan with abundant SOC, the warming effect remained as high as 26% °C^−1^ during four growing seasons, which is even more remarkable than the present results^33^. In our research site, no significant influence of soil warming treatment on SOC was confirmed. However, it is possible that a period of 6 years might be not enough to confirm the significant warming effect on SOC. Further continuous warming treatment with temporal analysis of SOC will be needed to accurately examine the long-term warming effect on SOC. In addition, not only the quantitative dynamics of SOC under warming, we should also note the soil warming effect on the SOC decomposition processes by microbiota because previous studies^22^,^23^,^24^ and our collaborative study (Kondo *et al*. *in preparation*) confirmed the shift of species composition of microbiota under soil warming.

The soil nitrogen content is also important for decomposition of organic carbon by the microbiota. Nitrogen and microbial biomass are positively correlated^43^, and decomposition of organic carbon will be difficult under nitrogen-limited conditions even if SOC is abundant. Concerning temperature sensitivity, it has been found that the *Q*_10_ of *R*_h_ was positively correlated with the nitrogen-to-carbon (N:C) ratio in soil^44^. This suggested that the *Q*_10_ of *R*_h_ is smaller under nitrogen-limited conditions. The nitrogen content to a depth of 30 cm at our research site was 0.69 kg m^−2^, and the average C:N ratio was 14.2. The limit of nitrogen mineralization and immobilization is thought to occur at a C:N ratio of 20^45^. Below this threshold, nitrogen mineralization is thought to occur. Thus, the soil nitrogen content and C:N ratio at our research site do not appear to have been nitrogen limited, so the nitrogen content was not a limiting factor for *R*_h_. These results suggest that the co-occurrence of abundant SOC and adequate nitrogen can explain why the stimulatory effect of soil warming was maintained throughout our study period.

At our forest site, we found a strong and marked exponential relationship between soil temperature and *F*_c_ in each year and in both the control and the warming treatment (Fig. 3a,b). In addition, the relationship between seasonally estimated *F*_c_ and soil temperature was strong, especially in dormant period (Fig. 2c). These results suggest that soil temperature is the primary environmental factor that controls the seasonal variation of *F*_c_, as was shown by Lloyd and Taylor^4^.

*Q*_10_ is reported to decrease with increasing temperature^46^,^47^. However, the *Q*_10_ of *R*_hw_ in our study did not differ significantly from that of *R*_h_, and we found no decreasing trend for *Q*_10_ after 6 years of the warming treatment. This result indicates that the thermal adaptation of temperature sensitivity did not occur. Schindlbacher *et al*.^41^ also showed that *Q*_10_ did not decrease after 9 years of warming treatment in a soil incubation experiment, and suggested that no thermal adaptation of *R*_h_ should be expected after long-term soil warming. We found no significant relationship between the mean annual soil temperature and raw-*Q*_10_, but found a significant negative relationship between the normalized-*Q*_10_ and the mean annual soil temperature for *R*_hw_. This indicates that the influence of the summer soil moisture on the interannual variation of *Q*_10_ is stronger than that of the mean annual soil temperature. However, it is difficult to confirm the direct influence of the mean annual soil temperature on the interannual variation of *Q*_10_ because of the small interannual variation of soil temperature (15.1 to 15.7 °C in the control, versus 17.5 to 18.4 °C in the warming treatment). The result only partly implies that an increase in the mean annual soil temperature could cause *Q*_10_ to decrease under a warmer climate.

According to the temperature-response model of Lloyd and Taylor^4^, the stimulatory effect of soil warming on soil *F*_c_ should decrease with increasing temperature. In our study, the seasonal trend of the warming effect peaked in the winter and decreased in the summer, and the simulated warming effect calculated from Lloyd and Taylor’s model showed the same seasonal trend. On the other hand, we found no significant relationship between the annual warming effect and the annual soil temperature. This may be because of the weak relationship between the mean annual soil temperature and the interannual variation of raw-*Q*_10_, which represents a stronger influence of soil moisture during the summer and a narrow range of annual soil temperatures. From these perspectives, the influence of soil temperature on the seasonal trend of the warming effect was clear, but the interannual pattern was more difficult to explain.

Overall, the relationship between soil moisture and *F*_c_ during the summer was not strong. However, that relationship was strong in all treatments in 2013 due to the low summer rainfall that year. When we considered the influence of soil moisture on the interannual trend for *F*_c_, we found a significant relationship between the estimated summer *F*_c_ and the seasonal mean soil moisture content for *R*_hw_. These results suggest that the influence of soil moisture on the seasonal variation of *F*_c_ was unclear, except during the summer drought, but that the influence was clear for the interannual variation of *F*_c_, especially for *R*_hw_. The warming treatment did not change the annual mean soil moisture content significantly at a depth of 10 cm, but the strong relationship between the estimated summer *F*_c_ and the seasonal mean soil moisture content for *R*_hw_ implies that the demand for water was relatively strong in the top 10 cm of the warmed soil.

Both the seasonal mean soil moisture content and the total summer precipitation were significantly related to raw-*Q*_10_ for *R*_h_ and *R*_hw_. The peak raw-*Q*_10_ for *R*_h_ was in 2011 (2.90). The soil moisture content and precipitation in that summer were 18.5% and 960 mm, respectively. Higher soil moisture content and precipitation led to a slight decrease in raw-*Q*_10_. A concave-down relationship between soil moisture content and the *Q*_10_ of *R*_h_ was shown in a previous study^28^. On the other hand, the peak raw-*Q*_10_ for *R*_hw_ was in 2012 (3.02), the year with the most precipitation (1187.5 mm). The raw-*Q*_10_ for *R*_hw_ in response to summer precipitation increased with high precipitation in summer, and did not show the decreasing trend along with the increase of summer precipitation (Fig. 5f). An increasing trend with increasing soil moisture was confirmed for *Q*_10_ of *R*_s_ in relatively arid environments^48^,^49^. This suggests that the difference in trends depends on whether the water supply available to support *F*_c_ was sufficient for the soil microbiota. From this perspective, the response of annual *Q*_10_ to summer precipitation can differ between *R*_h_ and *R*_hw_, and suggests higher water demand under soil warming than in the control during the summer. The estimated summer *F*_c_ values account for about 45% of the estimated annual values in each treatment at our forest site. Precipitation and the resulting increase in soil moisture content appear to strongly influence the annual temperature sensitivity by affecting the trend in summer *F*_c_.

We found a marginally significant relationship between the total summer precipitation and the annual soil warming effect. The different responses of the temperature sensitivity of *R*_h_ and *R*_hw_ to summer precipitation and the resulting change in soil moisture content appear to contribute to the annual variation in the warming effect. In addition, we found wide ranges of seasonal variation in total summer precipitation and soil moisture content. The summer precipitation ranged from 350.5 to 1187.5 mm during our study. The mean summer soil moisture content ranged from 12.1 to 22.2% in the control treatment and from 14.4 to 23.0% in the warming treatment. These wide ranges from dry to moist explain some of the annual variation in the warming effect.

There has been no information in the literature on the long-term effect of soil warming on *R*_h_ in warm-temperate forests. Using a multichannel automated chamber measurement system and infrared heater for warming the soil, we provide the first evidence that the stimulatory effect of soil warming on soil *R*_h_ is maintained for at least 6 years, and the magnitude of the effect was comparable to values predicted using a traditional temperature-response model. In addition, we found that the interannual variation in effect of soil warming on *R*_h_ was positively related to the total summer precipitation. Overall, our results suggest that the stimulatory effect of soil warming in an Asian monsoon forest with abundant SOC and a humid climate may be maintained for a longer period than was previously expected. Under the predicted future warmer and more humid conditions, the carbon sink strength of these forests will therefore potentially be weakened as a result of increased *R*_h_. Of course, the photosynthetic uptake of carbon by the trees may also increase, thereby balancing the soil carbon loss to some unknown extent. We will need further continuous *F*_c_ measurements to support a decade-level estimation and determine whether the results of the present study remain valid over longer time periods. In addition, it is necessary to conduct long-term warming experiments at other forest sites with a variety of soils to confirm how widely the present results can be generalized.

## Materials and Methods

### Site description

The study site is in a warm-temperate evergreen broad-leaved forest in the Miyazaki University Forest (31°51′N, 131°18′E; 130 m asl), Kyushu, southern Japan. The dominant species is *Castanopsis cuspidata* and *Machilus thunbergii* (about 55 years old). The forest’s understory was dominated by *Eurya japonica*. The tree density is about 1175 stems ha^−1^. We used climate records (2009 to 2014) from the Japan Meteorology Agency weather observation station in Miyazaki as reference data. The mean annual temperature was 17.6 °C, with monthly means ranging from 7.0 °C in January to 27.9 °C in August, and the mean annual precipitation was 2604 mm, with 70% of the total occurring during the growing season. The soil is a well-drained brown forest soil developed from volcaniclastic sediment, and the thicknesses of the A and B horizons were 20 and 40 cm, respectively. Soil pH in the upper 40 cm averaged 5.7, mildly acidic as commonly observed in Japanese forest soil.

### Chamber system

To obtain continuous soil CO_2_ flux measurements, we used a multichannel automated chamber system. The system was a modified version of the system described by Liang *et al*.^50^,^51^. The main components of this system were an infrared gas analyser (IRGA; LI-820, Li-Cor, Lincoln, NE, USA), a datalogger (CR1000, Campbell Scientific Inc., Logan, UT, USA), an air compressor (super oil-free BEBICON 0.2 LP-7s, Hitachi Ltd., Tokyo, Japan) and handmade 15 chambers. Square chambers (90 cm long × 90 cm wide × 50 cm tall) were made of a plastic-coated steel frame (30 mm × 30 mm) to which was affixed a clear PVC wall. They were connected to the control box with a power supply, thermocouples for measurement of air and soil temperatures (5 cm below the soil surface), and tubing for sampling and circulating the air. Thermocouples from these chambers were connected to the handmade multiplexer, and signals were output to the CR1000 datalogger. Soil moisture sensors (CS616, Campbell Scientific Inc.) were buried 10 cm below the soil surface within the chambers, and their signals were also recorded by the data logger. Each chamber was also connected to the compressed air for opening and closing of the chamber lid via an electric valve and two pneumatic cylinders (LAC-20B, CKD Corp., Nagoya, Japan) attached to the lid of each chamber. This enabled continuous and automatic opening and closing according to a program installed in the data logger. Only one chamber was closed at a time, during a flux measurement period that lasted for 4 min, then was re-opened after the measurement to allow measurement of the next chamber. By repeating this cycle, measurements in all chambers were completed within an hour. During each measurement, two embedded electric fans (MF12B, Nihon Blower Ltd., Tokyo, Japan) stirred the air inside the closed chamber to even out the CO_2_ concentration. Air inside the closed chamber was then passed through the IRGA in the control box with a micro-diaphragm pump (5.0 L min^−1^; CM-50, Enomoto Ltd., Tokyo, Japan). The data logger recorded signals from the IRGA and other sensors at 10-s intervals.

The system was first installed in December 2008. Before starting the warming treatment in January 2009, we established trenches around 10 of the 15 chambers to keep out roots, and then carefully clipped all understory vegetation growing inside the chamber thus excluding root respiration and letting us measure heterotrophic respiration (*R*_h_). During this operation, a root-cutting chainsaw was used to sever any roots in the soil to a depth of 40 cm. PVC plates (100 × 30 × 0.4 cm) were then inserted around each trenched chamber to prevent roots from growing into the chamber. Soil CO_2_ efflux (*F*_c_, μmol CO_2_ m^−2^ s^−1^) was measured for 19 days after the trenching treatment to measure *R*_h_ in those trenched 10 chambers before warming treatment. Thereafter, a warming treatment was set up for five of the trenched chambers (the other five were the control treatment). For the warming treatment, an infrared heater (Carbon lump heater, Sakaguchi E.H. VOC corp., Tokyo, Japan) was hung at the centre of each warmed chamber (*R*_hw_), about 1.6 m above the soil surface. Due to this warming treatment, soil temperature at a depth of 5 cm below the surface increased by about 2.5 °C above the soil temperature of control treatment. The trenching treatment was not applied to five additional chambers, which we used to measure total soil respiration (*R*_s_ = the sum of heterotrophic and autotrophic respiration).

### Data processing and analysis

*F*_c_ was calculated with the following equation:


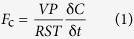


where, *V* is the chamber volume (m^3^), *P* is the air pressure (Pa), *R* is the ideal gas constant (8.314 Pa m^3^ K^−1^ mol^−1^), *S* is the soil surface area inside the chamber (m^2^), *T* is the air temperature (K) inside the chamber, and δC/δt is the rate of change of the CO_2_ mole fraction (μmol mol^−1^ s^−1^) calculated from the chamber data using the least-squares method and based on the assumption of a linear change during the measurement period. Before we calculated the average *F*_c_ in each treatment, we applied the Smirnov–Grubbs test (with significance at *p* < 0.1) for the average of *F*_c_ values during the 6 years for all chambers in each experimental group to remove outlier data that showed an unusual *F*_c_ pattern from the analysis.

To analyse the temperature response of each chamber and treatment, we used regression analysis following an exponential model:


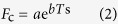


where *a* is the efflux at 0 °C, *b* is a curve-fitting parameter for temperature sensitivity, and *T*_s_ is the soil temperature (°C). From Equation (2), *Q*_10_ can be calculated to express the increase in soil respiration for every 10 °C temperature rise, as follows:


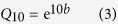


where *b* is the curve-fitting parameter from Equation 2. We used this equation to derive a mean value of *Q*_10_ from the annual temperature response curve of *F*_c_. In addition, we also calculated the soil-moisture-normalized *Q*_10_ values using *F*_c_ data when the soil moisture content was within the range of variation in the soil moisture (annual average ± 1 SD; hereafter, normalized-*Q*_10_). To examine the influence of warming on annual *Q*_10_, we used the two-tailed Student’s *t*-test for the relationship between *R*_h_ and *R*_hw_ (with significance at *p* < 0.05).

On the other hand, Lloyd and Taylor^4^ developed the following model to represent *F*_c_:


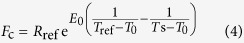


where *R*_ref_ (μmol CO_2_ m^−2^ s^−1^) is the soil CO_2_ efflux at a specified reference soil temperature, *E*_0_ is the coefficient of temperature sensitivity, *T*_ref_ is the specified reference soil temperature (288.15 K), *T*_0_ is the soil temperature when *R*_s_ is zero (227.13 K), and *T*_s_ is the observed soil temperature (K). We used this Equation (4) in our study to fill gaps in the data and when we ran the annual temperature-response equation of *F*_c_.

The number of gap days totalled 105 days during the 2202 days of measurement from December 2008 to December 2014, corresponding to 4.8% of the whole measurement period. The biggest gap was from 29 September to 31 October 2013 due to problem of the IRGA, and this was the only gap longer than 30 days during the 6 years of observations. We analysed the temperature-response of *F*_c_ for each chamber in every year. We used Equations (2) and (3) to calculate the *Q*_10_ values, and Equation (4) to estimate *F*_c_ for data gaps in each chamber. After the gap-filling, the estimated annual or seasonal fluxes (tC ha^−1^) were calculated for three seasons: the summer, from July to September; the growing period, from May to October; and the dormant period, from January to April and from November to December. To examine the influence of warming on the estimated fluxes, we performed the two-tailed Student’s *t*-test on the 6-year average value for each period to test for differences between *R*_h_ and *R*_hw_ (with significance at *p* < 0.05).

To avoid the confounding effects of soil temperature and soil moisture, we first subtracted the predicted values of *F*_c_ using Equation (4) from the observed values, and the resulting residual values (the temperature-normalized *F*_c_) were used to analyse the relationships between soil moisture and *F*_c_. To calculate the strength of the relationship, we used the following quadratic regression:


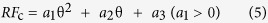


where *RF*_c_ (μmol CO_2_ m^−2^ s^−1^) means the temperature-normalized *F*_c_, θ is the volumetric soil moisture content (%), *a*_1,_ *a*_2_, and *a*_3_ are curve-fitting parameters. However, when the value of *a*_1_ in Equation (5) was 0 or negative, we used Equation (6) instead:
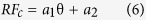


We used SigmaPlot 12.5 (Systat Software, San Jose, CA, USA) as our statistical software for our analyses.

### Analysis of the warming effect

To analyse the warming effect on *R*_h_, we first calculated treatment coefficients for *R*_h_ and *R*_hw_ based on 18 days of hourly *F*_c_ data from each chamber from 20 December 2008 to 6 January 2009 to modify the heterogeneity of the *F*_c_ value between *R*_h_ and *R*_hw_ before the start of the warming treatment in January 2009. We calculated the coefficients as *F*_c (all)_/*F*_c (treatment)_, where *F*_c (all)_ means the average of the hourly *F*_c_ in the trenched chambers (μmol CO_2_ m^−2^ s^−1^) during the 18 days before the warming treatment began, and *F*_c (treatment)_ represents the average hourly *F*_c_ for each treatment during the same period (*R*_h_ or *R*_hw_, μmol CO_2_ m^−2^ s^−1^). Those coefficients were calculated to be 0.9665 for *R*_h_ and 1.0359 for *R*_hw_, respectively. Thus, the warming effect on *F*_c_ (% °C^−1^) was calculated as (1.0359 *R*_hw_ − 0.9665 *R*_h_) × 100/[0.9665 *R*_h_ (*T*_sw_ − *T*_sc_)], where *T*_sc_ is the soil temperature in the *R*_h_ treatment (°C), and *T*_sw_ is the soil temperature in the *R*_hw_ treatment (°C). Modelled *F*_c_ values for *R*_h_ and *R*_hw_ were calculated by assigning the soil temperatures in the *R*_h_ and *R*_hw_ treatments to the annual temperature-response equation for *R*_h_ (Equation 4). There were two versions of the modelled *F*_c_: the ‘raw-*Q*_10_’ version (in which we used the all measured data without accounting for soil moisture anomalies) and the ‘normalized-*Q*_10_’ version. For the normalized-*Q*_10_ version, the annual temperature-response equation using Equation (4) for *R*_h_ was derived from the same range of soil moisture values that we used when calculating the normalized-*Q*_10_. Then, the modelled warming effect (% °C^−1^) was calculated for the two versions of the model as (*R*_h___*T*sw_ − *R*_h___*T*sc_) × 100/[*R*_h___*T*sc_ (*T*_sw_ − *T*_sc_)], where *R*_h_*T*sc_ is the modelled *F*_c_ of *R*_h_ (μmol CO_2_ m^−2^ s^−1^) and *R*_h_*T*sw_ is the modelled *F*_c_ of *R*_hw_ (μmol CO_2_ m^−2^ s^−1^) calculated by substituting soil temperature in *R*_hw_ to annual temperature-response equation of *R*_h_. We calculated the 31-day moving averages (from 15 days before to 15 days after a given date) from the observed daily warming effect and the two versions of the model. We also calculated the observed and modelled annual warming effects from the annual average of daily *F*_c_.

### Analysis of soil carbon and nitrogen

To analyse the soil organic carbon (SOC) and total nitrogen (TN) concentrations, we hammered three PVC tubes (10.7 cm in inner-diameter, 70 cm in length) into the soil to obtain samples to a depth of 30 cm in June 2014, about 1–2 m from three of the chambers. The soil cores were cut into six 5-cm segments for analysis, and those soil samples were dried in a 80 °C oven for 1 week. After weighting the dried soil samples, each segment was passed through a 2-mm sieve to remove coarse fragments and large roots. The dried soil samples were then ground into a fine powder with a mortar and further dried at 100 °C for 1 week. The SOC and TN contents were analysed using an NC analyser (FLASH EA 1112, Thermo Electron Corp., Waltham, MA, USA) with combustion at 900 °C. In the middle of August 2016, we also sampled small soil cores (5.0 cm in diameter) to a depth of 5 cm in three of the trenched and the warmed trenched chambers to examine the warming effect on SOC (warming treatment was still continued until that time). We performed small soil core sampling in that way to minimize soil disturbance in each chamber due to soil core sampling. The SOC contents in those small soil cores were analysed following the same procedure as the above. Difference in SOC between control and warming treatments was corrected on the assumption that SOC decreased lineally along with time due to soil warming treatment (from the start of warming treatment, early January in 2009) to estimate SOC content in warmed soil in the end of December 2014. We performed the two-tailed Student’s *t*-test on SOC content in each treatment to examine if there was significant effect of soil warming on SOC (with significance at *p* < 0.05).

## Additional Information

**How to cite this article**: Teramoto, M. *et al*. Sustained acceleration of soil carbon decomposition observed in a 6-year warming experiment in a warm-temperate forest in southern Japan. *Sci. Rep.* **6**, 35563; doi: 10.1038/srep35563 (2016).
